# Effect of Multi-Dose Dispensing on Medication Regimen Complexity: A Real-World Study

**DOI:** 10.3390/jcm13051205

**Published:** 2024-02-20

**Authors:** Sunmin Lee, Jongsung Hahn, Heungjo Kim, Min Jung Chang

**Affiliations:** 1 College of Pharmacy and Research Institute of Life and Pharmaceutical Sciences, Sunchon National University, Suncheon 57922, Republic of Korea; smlee@scnu.ac.kr; 2 Department of Pharmacy and Yonsei Institute of Pharmaceutical Sciences, College of Pharmacy, Yonsei University, Incheon 21983, Republic of Korea; heungjo@yonsei.ac.kr; 3 College of Pharmacy, Jeonbuk National University, Jeonju 54896, Republic of Korea; jongsung@jbnu.ac.kr; 4 Department of Pharmaceutical Medicine and Regulatory Science, Yonsei University, Incheon 21983, Republic of Korea; 5 Graduate Program of Industrial Pharmaceutical Science, Incheon 21983, Republic of Korea

**Keywords:** multi dose dispensing, medication regimen complexity, pharmacist practice, regimen complexity, dose administration aid

## Abstract

(1) Background: Older patients frequently require dosing aids, such as multi-dose medication dispensing (MMD) when they experience medication regimen complexity (MRC) with increased drug use. However, the evaluations of the efficacy of MMD alterations remain limited. (2) Methods: A total of 1120 patients were included in the study who were discharged from hospital during the study period of January to March 2019. The Medication Regimen Complexity Index (MRCI) score, a validated 65-item tool in Korea (MRCI-K), was used to quantify MRC. The original MRCI-K scores, representing the typical administration based on prescription information, were compared to recalculated MRCI-K scores measured following MMD during the hospital dispensing period. Differences in MRCI-K across the top four wards based on the numbers of discharge prescription medications were assessed, and the overall scores were categorized into quartiles to identify MMD’s impact within each group. We confirmed the effect of MMD based on the patient’s admission diagnosis depending on MRCI. (3) Results: The mean (standard deviation) of original MRCI scores was 26.2 (13.4), which decreased to 18.9 (8.8) after applying MMD. The decrease in MRCI scores after MMD was statistically significant in all four wards, with the Orthopedic Surgery ward showing the biggest decrease. The patients with MRCI scores in the highest quartile group demonstrated the greatest improvement as a result of the implementation of MMD. Respiratory diseases exhibited the highest baseline MRCI scores due to formulation complexity, and ear, nose, and throat patients demonstrated the most significant reduction in MRC after MMD, depending on the diagnostic criteria at administration. (4) Conclusions: We confirmed the reduction in MRC after applying MMD, as a significant decrease in MRCI-K scores. This study highlights the need to deliver effective pharmacist-led services to identify patients who would benefit from MMD.

## 1. Introduction

The expanding demographic of older patients with multiple comorbidities has raised concerns over increasing numbers of drugs, which are associated with increasingly complex medication regimens [1]. Medication regimen complexity (MRC) arises from factors such as diverse dosage forms, dosing frequencies, and administration methods, which may not directly correlate with the number of medications. MRC has been identified as a significant risk factor that might cause drug-related problems, and particularly is recognized as the primary cause of unintentional medication non-adherence [2]. Intervention involves simplifying MRC without altering the therapeutic regimen of pharmacotherapy since MRC could cause a practical problem for vulnerable patients who require multiple medications [3].

Several attempts have been made to manage MRC, and one of the primary approaches has been the quantification of MRC. In 2004, George et al. developed the Medication Regimen Complexity Index (MRCI), a scoring instrument designed to assess the properties of MRC beyond the number of medications [4,5]. The MRCI comprises three sections and are as follows: dosage form (section A), dose frequency (section B), and additional instructions (section C). It assigns a score to each prescription, with the total score being the sum of the scores for each section [5]. Thus far, this instrument has been validated in various countries including USA, Germany, and Korea [6,7,8,9] and is widely used for the evaluation and in the research of MRC [4].

Dose administration aids individual medicine doses to be organized according to the prescribed dose schedule [10]. A multi-dose medication dispensing (MMD) system, known as an automated dispensing system, is one of the dose administrations that provides patients with unit doses of prescribed medications to enable them to take their medications at the same time. MMD can prepare all drugs to be taken at the same administration time in a disposable bag for patients administered polypharmacy, and is widely used worldwide including in Korea [11].

To date, several studies have examined which patients may benefit most from MMD. Despite the lack of clear indications for identifying who will benefit from MMD, its use is increasing due to its benefits, including improved medication safety, positive effects on medication adherence, and reductions in unused drug wastage [12,13,14]. However, few studies exist on the effectiveness of MMD in regard to effective MRC reduction.

Given that a significant portion of prescribing complexity is due to the increased frequency of drug administration [12], it is important to identify patient populations that would benefit most from reducing prescribing complexity through MMD. The purpose of this study was to evaluate MRC effectiveness with the discharge medications of patients admitted in a hospital, determine whether MRC can be lowered through MMD, and identify the patient group that experienced the greatest improvement.

## 2. Methods

### 2.1. Study Design and Patients

This was a descriptive, cross-sectional study at a 900-bed university-affiliated teaching hospital in Incheon, Korea. We included patients who received more than one medication per day as a discharge prescription from January to March 2019. Patients were excluded from the study if they were admitted to palliative care, chemotherapy, or intensive care units or were under 18 years of age. To identify the differences in MRCI-K according to the hospital ward, we selected the top four wards with the highest number of patients, meeting the inclusion criteria and randomly selecting 280 patients per ward (1120 patients in total), according to the G-power (effect size: 0.1; Type 1 error: 0.05: Type 2 error: 0.8). The parent study was approved by the Inha hospital Review Board (approval no. IRB 2022-05-002).

### 2.2. Data Collection

We collected patient demographic details, diagnosis according to the International Classification of Diseases 9th edition-Clinical Modification (ICD-10th-CM), length of stay, numbers of additional diagnoses excluding the primary diagnosis, and destination after discharge from the hospital’s electronic medical record system (EMR). We collected patient medication lists, which recorded not only discharge prescriptions and those taken during the hospital stay but also self-administered medication before admission. If patients were discharged after readmission, the records from the prior admissions were also collected.

### 2.3. Study Outcome

The primary endpoint of our study was to compare MRCI-K score improvement across four wards after the implementation of MMD. We analyzed prescriptions for patients in the four wards using MRCI-K to confirm changes in scores after the application of MMD. Additionally, we grouped patients according to their primary diagnosis and identified the group with the greatest MRCI improvement based on the distribution of MRCI-K scores.

#### Multi-Dose Dispensing (MMD)

The hospital pharmacy is responsible for the MMD process, which includes the preparation and dispensing of medications. MMDs are adherence aids for oral medications, automatically machine-dispensed in disposable plastic bags according to instructions in hospital pharmacies. If the doctor orders the medication to be taken at the same time as the prescription instructions, the medication will be dispensed in the same plastic bag so that the patient can take them together. Half pills or multiple doses will be dispensed by the hospital pharmacy so that the doses can be taken in the same order.

### 2.4. MRCI Analysis

To quantify discharged medication complexity, we used MRCI score, which is a validated 65-item tool in Korea (MRCI-K). The MRCI-K measure was validated for validity and reliability in 2019 on patients discharged from the same hospital in the study [8]. The calculations were consistent with the original MRCI in that it consisted of scoring each medication on dosage form (section A), dose frequency (section B), and instructions (section C) aspects expressed as the aggregate of each section. Medication complexity was evaluated in two stages. The first was the original MRCI-K, which did not consider MMD, only usual administration based on prescription information. Each section was scored based on the discharge medication regimen of the eligible patients.

**Figure 1:**
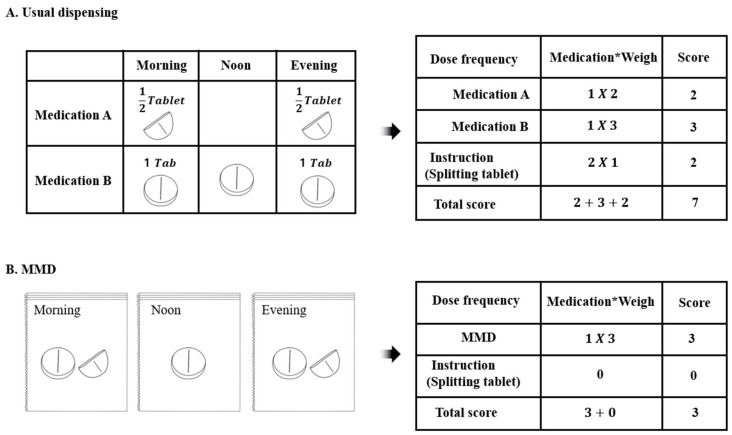
An example of measuring regimen complexity using MRCI-K between a usual dispensing and MMD.

Second, we also assessed the MRCI-K score to evaluate regimen complexity change after MMD during the hospital dispensing period. To evaluate dosing frequency after MMD, we recalculated the dose frequency section scores because the dispensing process simplified the schedule by matching the administration time according to matching instructions, while the dosage form did not change. In addition, we rescored section C because the automatic tablet system can lead to preparing multiple doses and pill splitting and sorting them into pouches to reduce regimen complexity. The MRCI-K score was calculated using an Excel formula by two independent researchers. Figure 1 provides an example of measuring prescription complexity using MRCI-K. Prescription complexity was calculated by considering factors such as the number of drug doses and instructions for splitting the drug, while maintaining a constant dosage form for all drugs. However, after dispensing, if two or more drugs were administered together at the same time, the number of doses varied depending on the dispensing process and the prescription instructions were not considered complex due to dispensing.

### 2.5. Statistical Analysis

Descriptive statistics were expressed as frequencies for categorical variables and means and standard deviations for variables measured on a continuous scale. We classified MRCI-K scores by quartile based on total MRCI-K score distribution. The normality of the distribution was confirmed using histograms to assess kurtosis and skewness. Factors that were not normally distributed were expressed as median and quartile values. To confirm the effect of regimen simplification after MMD, score differences in MRCI-K were identified according to admission ward, quartile group, and diagnosis at admission. In addition, score changes with MMD application across the groups were evaluated. Differences between scores were evaluated using paired *t*-tests, Wilcoxon signed rank tests, or ANOVAs. Statistical significance was set as a two-sided *p*-value. Statistical analyses were performed using IBM SPSS Statistics version 21.0 (IBM Corp. Released 2012. IBM SPSS Statistics for Windows, Version 21.0. Armonk, NY, USA)

MRCI-K is the Korean version of medication regimen complexity index, and MMD is multi-dose medication dispensing.

## 3. Results

**Figure 2:**
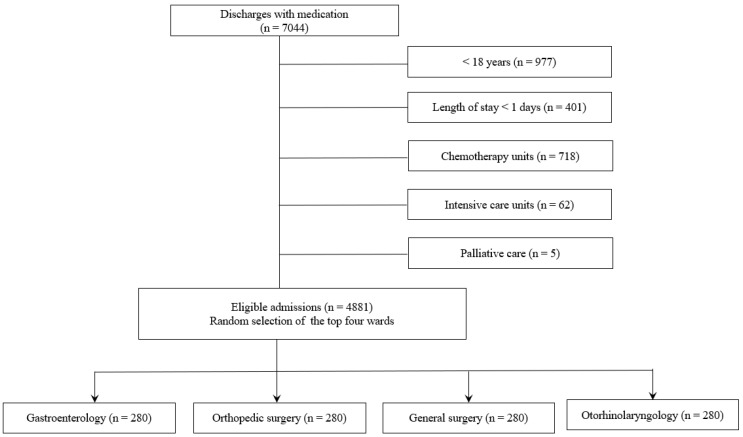
Case flow chart.

We included 240 patients each from the gastroenterology, orthopedics, general surgery, and otorhinolaryngology wards who were hospitalized for longer than 24 h with discharge medications (Figure 2).

The characteristics of patients included in this study are presented in Table 1. Female patients comprised 40.9% of all patients, and the mean age was 58.8 ± 18.6 years. The patients in the gastroenterology ward exhibited the highest mean age of 67.1 years, with the otorhinolaryngology having the lowest mean age of 51.3 years. Primary diagnoses were categorized according to the ICD-10th-CM. Gastrointestinal diseases were the most prevalent primary admission diagnoses, accounting for 28.2% of cases, followed by neoplasms at 23.2%, injury, poisoning, and certain other external causes at 12.5%, and respiratory system-related conditions at 10.0%. Additionally, patients had an average of 1.0 extra diagnoses in addition to their primary admission diagnosis. Most patients were discharged to their homes, constituting 92.3% of the total discharges. The overall number of drugs was 5.7 ± 3.2. Significant differences in MRCI-K sections were observed among the four wards, with otorhinolaryngology showing high scores in all sections of the MRCI-K. Patient characteristics varied among the four different wards (Table 1).

After applying MMD, the MRCI showed an overall reduction from 26.2 ± 13.4 to 18.9 ± 8.8 (Supplementary Table S1). This decrease in MRCI scores was primarily attributed to changes in section B (dose frequency) rather than section C (instructions). Orthopedic surgery and otorhinolaryngology showed substantial reductions in scores, decreasing from 25.5 ± 12.6 to 17.0 ± 8.1 and from 31.1 ± 11.9 to 22.6 ± 8, respectively. These decreases were mainly due to a decrease in the dose frequency following the implementation of MMD. A detailed break-down of scores is provided in Table 2.

**Figure 3:**
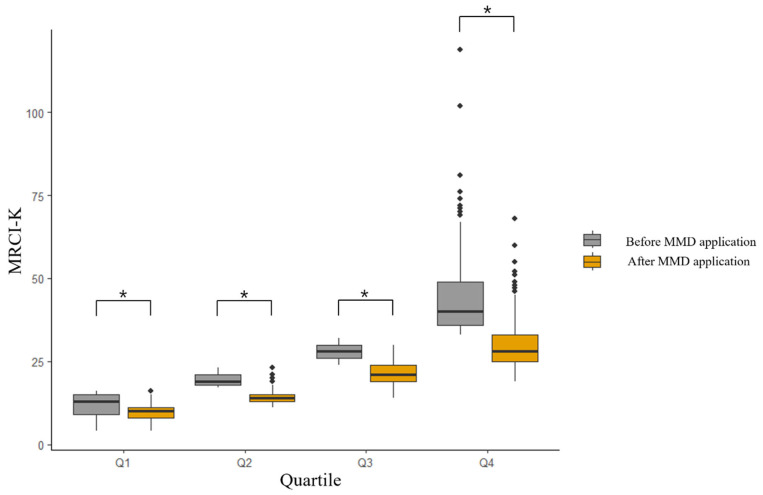
Decreases in Medication Regimen Complexity Index in Korea (MRCI-K) after multi-dose dispensing across the quartiles. * *p*-Value < 0.001.

When classifying MRCI-K scores into quartiles, Q1 is marked by a threshold of 17 points, while Q4 was defined by 33 points. The median number of medications (with interquartile range, IQR) was 5 (4–7). In each section of the MRCI-K distribution, the median dosing frequencies and instructions significantly increased, and this corresponded to higher MRCI scores (Supplementary Table S2). Following the implementation of MMD, notable reductions in complexity were observed, with section B showing the most significant decrease. Q4 had the largest decrease of −11.3, while Q2 had a decrease of −6.3, Q3 of −0.4.8, and Q1 of −2.2. Additionally, this reduction was most pronounced in the highest quartile of MRCI-K scores (−13.8 (5.8), *p* < 0.001; Figure 3).

**Figure 4:**
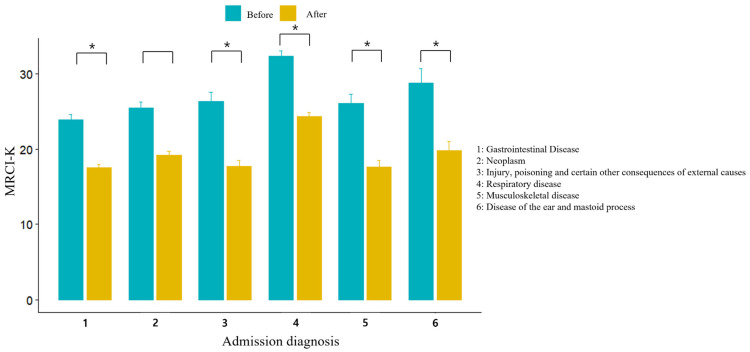
Decreases in Medication Regimen Complexity according to primary diagnosis at admission after the application of multi-dose dispensing (MMD). * *p*-Value < 0.001.

Figure 4 shows the reduced MRCI with the six major primary diagnoses at admission. Among the primary diagnoses at admission, respiratory system diseases showed the highest MRCI, followed by diseases of the ear and mastoid process, neoplasms, musculoskeletal system and connective tissue diseases, digestive system diseases, and injury, poisoning, and certain other consequences of external causes, respectively. The order of scores for the major primary diagnoses was the same after MMD application (Supplementary Table S3). Moreover, the MRCI after MMD application significantly decreased in all groups, with the greatest decreases in those with diseases of the ear and mastoid processes and the respiratory system (−8.9 (6.0), *p* < 0.001) due to reductions in both dose frequency and instructions section.

## 4. Discussion

This study demonstrated that MMD significantly reduced MRC in pharmacy-led dispensing across all wards, irrespective of the therapeutic regimen. The reduction was particularly notable in dose frequency and in prescription instructions. This is the first study to use the MRCI to explore the effectiveness of MMD in hospitalized patients at discharge. The MRCI was proposed and developed as a gold-standard scoring system for quantifying MRC according to prescription formulation, dose frequency, and instructions [4].

This research may have practical applications, enabling tailored drug management strategies for specific patient populations. First, MRC can vary greatly depending on the patient’s hospitalization ward, and the effectiveness of MMD can also vary greatly. Our study showed that dosage form resulted in increased MRC among patients in the otorhinolaryngology ward, and the complexity decreased in total following MMD application, with a notable decrease in dose frequency in particular. This result suggests that, even in cases of high formulation complexity, the effectiveness of regimen simplification can be maximized by pharmacy-led MMD interventions without alterations in therapeutic regimens during pharmacotherapy.

Second, a reduction in MRC was observed across all complexity-based quartile groups, with the most substantial effect in the highest quartile (Q4). Thus, higher MRC levels necessitate the simplification of regimen complexity, and active intervention can lead to a maximal reduction in MRC.

Third, we assessed MRC using the MRCI depending on patient primary admission diagnosis. In this study, the medications prescribed for respiratory system diseases exhibited the highest complexity. The dosage form was identified as a contributor to regimen complexity in treatments for respiratory system diseases, and the effectiveness of MMD was limited in this context. However, the simplification of regimen could be achieved after the implementation of MMD to improve regimen complexity. These findings suggest that pharmacies can assist in identifying patient groups that may benefit from MMD by scoring MRCI upon admission and quantifying the effectiveness of MMD.

Hospitalization leads to changes in medication use in patients, and an increase in the number of medications increases MRC. In our study, MRC on discharge was less complex than in previous studies, which included hospitalized patients in other disease groups. When the quartile of the complexity score was checked, the highest was 33 points, which is lower than in previous studies [13,14,15]. According to primary diagnosis at admission, patients with respiratory diseases had the highest complexity on discharge. The findings of the current study are consistent with previous studies showing that respiratory diseases were associated with therapeutic complexity in polypathological patients hospitalized in internal medicine wards [16]. Additionally, a previous study found that MRC has an association with increased dosage form and respiratory disease, indicating that these factors affect a patient’s perception of MRC [17]; these results agree with the findings of our study. Therefore, complex formulations evidently contribute to high MRC, and further research is needed to manage prescription complexity for respiratory diseases and should target patients eligible for intervention or those with significant regimen complexity.

In most previous studies, the MRCI has been used for the assessment of MRC for a range of patients with various diseases [18]. This instrument has been applied not only for the evaluation of MRC of whole medications, but also for intervention studies that can positively affect patient pharmacotherapy [19,20]. Most studies referring to the effect of dose frequency on the MRCI focus on interventions to simplify the MRC [20,21]. Our study involved a high number of additional instructions (section C) based on EMR records. To confirm the effectiveness of the MMD, which involved packaging the same administered prescriptions as one-time doses, we recalculated the MRCI after applying MMD and found a significant reduction in MRC due to the reduced number of dose frequencies after packaging. Further, scores decreased as the number of instructions decreased because the process of packing multiple medications into a pack, splitting the pills, or dispensing them into powder form was completed during the dispensing process. This was understood to be a simplification of the patient’s medication administration process that alleviates the burden of MRC. Consequently, in interventional studies, MMD effectively reduced scores compared to interventions involving the general administration process, such as changing prescription through a doctor [19,22]. Therefore, the pharmacy-led dispensing process is efficient in simplifying the intervention process, irrespective of therapeutic complexity.

Previous studies have reported that pharmacists’ role in simplification regimens is limited to being data coders or data analysts, and few studies of interventions have examined differences in scores [18]. Our results suggest that further research is needed to identify the role of pharmacists in the dispensing process as a difference in MRCI-K scores to determine the effectiveness of MMDs and differences in clinical outcomes.

This study is the first to confirm the effectiveness of MMD using MRCI. However, this study has some limitations. MRC might have been underestimated due to missing records, although pre-admission medications were required to be recorded electronically in our hospital system for all patients. In addition, this study is a retrospective, single-center study, and a multicenter, larger study is required to generalize the results and confirm our conclusions in various clinical practices.

In conclusion, when applying MMD to patients administered polypharmacy, the MRCI can be utilized to measure the impact of dosing aid services and objectively estimate the effectiveness of the intervention on individual patients. Additional research is necessary to determine the risk factors that contribute to increased prescriptions in different institutions with unique clinical practices and to develop tailored strategies accordingly. Furthermore, a comprehensive evaluation of the multifaceted effectiveness of MMD, including an assessment of the economic benefits, should be conducted in a follow-up study.

## Figures and Tables

**Table 1:** Characteristics of patients included in this study.

	Medical Wards					
Variables	Total (n = 1120)	Gastroenterology (n = 280)	Orthopedic Surgery (n = 280)	General Surgery (n = 280)	Otorhinolaryngology (n = 280)	*p*-Value
Age, years, mean ± SD	58.8 ± 18.6	67.1 ± 15.4	56.8 ± 20.0	59.9 ± 17.0	51.3 ± 18.1	<0.001
Sex female, n (%)	459 (40.9)	99 (35.3)	133 (47.5)	113 (40.3)	114 (40.7)	0.03
Primary diagnosis, n (%)						
Gastrointestinal diseases	316 (28.2)	124 (44.2)	0 (0.0)	173 (61.7)	19 (6.7)	<0.001
Neoplasms	260 (23.2)	128 (45.7)	12 (4.2)	76 (27.1)	44 (15.7)	<0.001
Injury, poisoning, and certain other consequences of external causes	141 (12.5)	1 (0.3)	130 (46.4)	8 (2.8)	2 (0.7)	<0.001
Respiratory diseases	112 (10.0)	0 (0.0)	0 (0.0)	0 (0.0)	112 (40.0)	<0.001
Musculoskeletal diseases	88 (7.8)	0 (0.0)	88 (31.4)	0 (0.0)	0 (0.0)	<0.001
Ear, nose, and throat diseases	62 (5.5)	0 (0.0)	0 (0.0)	0 (0.0)	62 (22.1)	<0.001
Skin and subcutaneous tissue diseases	22 (1.9)	0 (0.0)	7 (2.5)	1 (0.3)	14 (5.0)	<0.001
Cardiovascular diseases	18 (1.6)	4 (1.4)	1 (0.3)	13 (4.6)	0 (0.0)	<0.001
Endocrine, nutritional, and metabolic diseases	5 (0.4)	2 (0.7)	3 (1.0)	0 (0.0)	0 (0.0)	<0.001
Others	96 (8.9)	21 (7.7)	39 (14.2)	9 (3.5)	27 (3.5)	<0.001
Length of stay (days), mean ± SD	7.2 ± 7.9	6.1 ± 5.2	8.3 ± 8.9	9.9 ± 10.3	4.5 ± 4.9	<0.001
Number of diagnoses except for principal diagnosis *, mean ± SD	1.0 ± 1.5	2.0 ± 1.8	0.7 ± 1.2	0.9 ± 1.6	0.5 ± 0.8	<0.001
Destination after discharge, n (%)						
Home	1034 (92.3)	267 (95.4)	217 (77.5)	273 (97.5)	277 (98.9)	<0.001
Transferred to another hospital	64 (5.7)	10 (3.5)	51 (18.2)	2 (0.714)	1 (0.357)	<0.001
Nursing home	22 (1.9)	3 (1.0)	12 (4.2)	5 (1.7)	2 (0.7)	<0.001
Number of medication	5.7 ± 3.2	5.8 ± 3.8	5.9 ± 3.4	5.1 ± 2.8	5.8 ± 2.6	0.021
Section A	2.4 ± 1.9	2.3 ± 1.5	1.6 ± 1.2	1.3 ± 0.8	4.5 ± 2.2	<0.001
Section B	11.2 ± 5.6	11.5 ± 7.1	11.2 ± 5.2	10.0 ± 4.7	12.1 ± 4.6	<0.001
Section C	12.5 ± 7.4	12.3 ± 8.5	12.6 ± 7.3	10.6 ± 6.0	14.5 ± 7.1	<0.001
A + B + C	26.2 ± 13.4	26.2 ± 16.1	25.5 ± 12.6	21.9 ± 10.6	31.1 ± 11.9	<0.001

* These diagnoses were categorized according to the ICD-10th-CM coding system.

**Table 2:** Decreases in the Medication Regimen Complexity Index in Korea (MRCI-K) after multi-dose dispensing (MMD) among four hospital wards. MRCI-K, the Korean version of medication regimen complexity index; MMD, multi-dose dispensing.

Wards	Dose Frequency	Instructions	MRCI-K	*p*-Value
Total	−6.2 (4.6)	−0.8 (1.3)	−7.3(5.4)	<0.001
Gastrointestinal medicine	−5.4 (5.6)	−0.8 (1.4)	−6.2 (6.5)	
Orthopedic surgery	−7.6 (4.7)	−0.8 (0.8)	−8.5 (5.1)	
General surgery	−5.6 (4.2)	−0.3 (0.8)	−6 (4.6)	
Otorhinolaryngology	−6.3 (3.4)	−1.2 (1.9)	−8.4 (4.8)	

## Data Availability

The data that support the findings of this study are not publicly available due to privacy or ethical restrictions.

## Supplementary Materials

The following supporting information can be downloaded at: https://www.mdpi.com/article/10.3390/jcm13051205/s1, Table S1: Comparisons of Medication Regimen Complexity Index in Korea (MRCI-K) after multi-dose dispensing (MMD) among four departments.; Table S2: Reduction of Medication Regimen Complexity Index in Korea (MRCI-K) according to ranges after the application of multi-dose dispensing (MMD); Table S3: Reduction of Medication Regimen Complexity according to primary diagnosis at admission after the application of multidose dispensing.
