# Supplementary material for: Effect of Multi-Dose Dispensing on Medication Regimen Complexity: A Real-World Study
Source: J Clin Med. 2024 Feb 20;13(5):1205. doi: 10.3390/jcm13051205 (PMC10932204; doi:10.3390/jcm13051205)
Supplement: Supplementary file 1 [file jcm-13-01205-s001.zip › jcm-2843690-supplementary.pdf]

Supplement Table S1. Comparisons of Medication Regimen Complexity Index in Korea (MRCI-K) after multi-dose dispensing (MMD) among four departments.

Variables	Total (n=1120)		Gastrointestinal medicine (n=280)		Orthopedic Surgery(n=280)		General Surgery (n=280)		Otorhinolaryngology (n=280)		p-value
Number of Medication	5.7 (3.2)		5.8 (3.8)		5.9 (3.4)		5.1 (2.8)		5.8 (2.6)		0.021
MMD application	No	Yes	No	Yes	No	Yes	No	Yes	No	Yes	
Section A	2.4 (1.9)	2.4 (1.9)	2.3 (1.5)	2.3 (1.5)	1.6 (1.2)	1.6 (1.2)	1.3 (0.8)	1.3 (0.8)	4.5 (2.2)	4.5 (2.2)	NA
Section B	11.2 (5.6)	4.9 (2.4)	11.5 (7.1)	6.0 (3.1)	11.2 (5.2)	3.6 (1.3)	10.0 (4.7)	4.3 (1.4)	12.1 (4.6)	5.7 (2.4)	0.001
Section C	12.5 (7.4)	11.7 (6.7)	12.3 (8.5)	11.5 (7.7)	12.6 (7.3)	11.8 (7.0)	10.6 (6.0)	10.2 (5.5)	14.5 (7.1)	13.2 (6.1)	0.001
A+ B + C	26.2 (13.4)	18.9 (8.8)	26.2 (16.1)	19.9 (10.6)	25.5 (12.6)	17.0 (8.1)	21.9 (10.6)	15. 9(6.5)	31.1 (11.9)	22.6 (8.1)	0.001

Data are presented as the mean (SD).

* Statistical analysis was conducted using paired T-tests and ANOVA tests.

MRCI-K, the Korean version of medication regimen complexity index; MMD, multi-dose dispensing

Supplement Table S2. Reduction of Medication Regimen Complexity Index in Korea (MRCI-K) according to ranges after the application of multi-dose dispensing (MMD)

**Quartile**†	**MRCI-K**‡		**Section A**		**Section B**		**Section C**		**p-value***
	Before	After	Before	After	Before	After	Before	After	
Q1	13 (9-15)	10 (8-11)	1 (1-1)	1 (1-1)	6 (4-7)	3 (3-4)	6 (4-7)	6 (4-6)	<0.001
Q2	19 (18-21)	14 (13-15)	1 (1-1)	1 (1-1)	9 (9-10)	4 (3-5)	9 (8-10)	8 (8-10)	<0.001
Q3	28 (26-30)	21 (19-24)	3 (1-5)	3 (1-5)	13 (11-13)	6 (4-7)	13 (11-14)	12 (10-13)	<0.001
Q4	40 (36-49)	28 (25-33)	3 (1-6)	3 (1-6)	17 (14-21)	6 (4-8)	21 (18-26)	19 (17-24)	<0.001
Total range	24 (17-33)	18 (12-24)	1 (1-3)	1 (1-3)	10 (8-14)	4 (3-6)	11 (8-16)	10 (8-14)	<0.001

Data are presented as the median (IQR).

†The MRCI-K range of each section was divided based on the time before MMD and corresponded to 1~16, 17~23, 24~32, and 33 or more, respectively.

‡MRCI-K= Section A (dosage) + Section B (dose frequency) + Section C (instruction).

*p-value of the difference in MRCI-K before and after MMD implementation.

MRCI-K, the Korean version of medication regimen complexity index; MMD, multi-dose dispensing.

Supplement Table S3. Reduction of Medication Regimen Complexity according to primary diagnosis at admission after the application of multidose dispensing

Disease category†	MRCI-K		Section A		Section B		Section C		p-value*
	Before	After	Before	After	Before	After	Before	After	
Gastrointestinal Diseases	23.9 (13.1)	17.5 (8.4)	1.9 (1.5)	1.9 (1.5)	10.5 (5.7)	4.9 (2.3)	11.5 (7.2)	10.9 (6.4)	<0.001
Neoplasms	25.4 (12.4)	19.2 (8.7)	2.3 (1.6)	2.3 (1.6)	11.2 (5.5)	5.6 (2.8)	11.9 (6.5)	11.3 (6.1)	<0.001
Injury, poisoning and certain other consequences of external causes	26.3 (14.6)	17.7 (9.2)	1.7 (1.1)	1.7 (1.1)	11.5 (6.1)	3.7 (1.4)	13.2 (8.4)	12.4 (8.1)	<0.001
Respiratory diseases	32.4 (7.0)	24.4 (4.9)	6.1 (1.6)	6.1 (1.6)	12.0 (2.4)	6.1 (2.0)	14.4 (4.6)	13.4 (3.9)	<0.001
Musculoskeletal diseases	26.1 (11.3)	17.6 (7.5)	1.8 (1.6)	1.8 (1.6)	11.4 (4.6)	3.8 (1.5)	12.9 (6.4)	12.1 (6.2)	<0.001
Diseases of the ear and mastoid process	28.8 (15.4)	19.8 (9.6)	2.5 (1.1)	2.5 (1.1)	12.9 (6.2)	5.8 (2.5)	13.4 (8.9)	11.5 (6.8)	<0.001

Data are presented as the mean (SD).

†Specified diagnoses were categorized according to the ICD-10th-CM coding system.

*p-value of the difference in MRCI-K before and after MMD implementation.
